# Nutrient uptake and assimilation in fragrant rosewood (*Dalbergia odorifera* T.C. Chen) seedlings in growing media with un-composted spent mushroom residue

**DOI:** 10.1371/journal.pone.0249534

**Published:** 2021-04-06

**Authors:** Xiaowen Li, Haitao Xia, Jinwang Wang, Qiuxia Chen

**Affiliations:** Zhejiang Institute of Subtropical Crops, Zhejiang Academy of Agricultural Sciences, Wenzhou, China; Universidade de Santiago de Compostela, SPAIN

## Abstract

The purpose of this study was to detect nutrient uptake and assimilation in woody plants subjected to growing media with some peat replaced by spent mushroom residue (SMR). Fragrant rosewood (*Dalbergia odorifera* T.C. Chen) seedlings were cultured in five types of growing media with SMR and peat in volumetric proportions of 0% (control), 25%, 50%, 75%, and 100%. With the increase of SMR proportion, ammonium- and nitrate-nitrogen (N) concentrations declined but available phosphorus (P) concentration and electrical conductance both increased. Seedlings in the full SMR substrates showed obvious mortality. Seedlings in substrates with SMR in proportions higher than 25% showed symptoms of excessive N and P toxicities. The utilization efficiency for P was highest in the 25% SMR growing-media. Mineral N in substrates had a positive relationship with growth and biomass but not with glutamine synthetase activity. Available P was negatively related with acid phosphatase activity in both leaves and roots. Un-composted SMR can replace 25% of peat in growing media for fragrant rosewood seedlings, benefitting P uptake and assimilation.

## Introduction

Population increase and environment pollution together provoke the replacement of plant production in traditional farmlands with that from a plant factory [1]. The soilless substrate is the most important substance for plant production in factory seedling cultivation. The growing media with high quality substrates can give plants a fertile environment where unexpected contamination is avoided [2]. Peat accounts for the main proportion of growing media for factory plant cultivation, but this substrate is not sustainable because it is a non-renewable resource. Alternative materials are greatly needed to replace peat in growing media.

Due to terrific flavor and aroma and significant nutritional and therapeutic values, yields of mushroom production have increased dramatically all over the world [3]. The global mushroom market size was valued at 12.74 million tons in 2018 and will reach 20.84 million tons by 2026, exhibiting a compound annual growth rate of 6.47% in the forecast period [4]. The mushroom cultivation market is estimated to account for USD 16.7 billion in 2020 [5]. The increasing production of mushrooms results in a large amount of spent mushroom residue (SMR) [6]. A gross amount of 2.5–5.0 kg SMR is produced from 1 kg of mushroom production [1,7,8]. Data in 2016 reported that a total of 0.2, 0.99, and 38.93 million tons of SMR had been accumulated in Turkey, Spain, and China, respectively [9]. Continuous accumulation of SMR generates piles of solid wastes that require significant cost for disposal. The replacement of peat in the growing media is a critical requirement for the soilless culture of horticultural plants. SMR is considered to be an excellent candidate for the replacement of peat as substrates in growing media [6,10,11].

SMR contains a considerable amount of nutritive compounds, such as nitrogen (N) and phosphorus (P), that can be reused as fertilizer sources [12]. These nutritional compounds result from metabolic activity of the fungus and can be extracted and used for producing biofertilizers [13]. Because of this quality, SMR was suggested as a replacement of peat for use in growing media [6,11,14]. Compared to spent residues that were composted by cork, olive husk, or cotton gin trash, that from mushroom cultivation and peat provided gerber (*Gerbera jamesonii* H. Bolus) with larger amounts of dry mass and higher flower yield [10]. However, the use of SMR in growing media is restricted by high saline toxicity for horticultural plants [6,10–12]. Growing media with SMR and peat was found to vary across plant species [6,15] or mushroom species [11]. Therefore, it was suggested to test the proportional replacement of peat by SMR for specific plant response when factors of mushroom species are given [12].

The proportion of SMR mixed with peat in growing media can balance the trait between salinity and nutrition. The 75% replacement of peat by SMR was used for seed germination for courgetti (*Cucurbita pepo* L. var. *Afrodite F1*), pepper (*Capsicum annum* L. var. *Lamuyo F1*), and tomato (*Lycopersicon esculentum* var. *Muchamiel*) plants [6]. A lower proportion of 50% replacement resulted in the largest amounts of dry matter and flower yield in gerber [10]. Furthermore, lower 20–50% replacement produces tomato and pepper seedlings with higher morphology [15]. The proportion of 25% replacement was found to be excellent in growing media for growth and nutrient assimilation in horticultural plants [12,16]. The growing media containing 25% fresh SMR has also been successfully used for the seedling cultivation for trees (*Pinus koraiensis*) [16] and shrubs (*Aralia elata*) [17]. A low proportion of 10% replacement was found to benefit growth and dry mass in lettuce (*Lactuca sativa*) [11]. The extent to which plants resist salinity and absorb nutrients is the major concern that determines the use of SMR proportion in growing media. The response to salinity can be assessed through mortality and growth but nutrient uptake and assimilation have not been well documented.

Fragrant rosewood (*Dalbergia odorifera* T.C. Chen) is a perennial legume tree that naturally distributes in subtropical and tropical areas of China [18,19]. Fragrant rosewood is a highly valued horticultural wood with a significant number of compounds that have medicinal usage [20,21]. Traditionally, fragrant rosewood is derived from heartwood in plantations [22], seedlings are mostly cultured in containers filled with peat and perlite in growing media [18,19]. Recent findings revealed that fragrant rosewood seedlings were sensitive to the change of growing media types made up of soil and organic residues [23]. This stirs further interest in detecting its response to growing media of SMR and peat. A solid substrate can guarantee the quality of fragrant rosewood seedlings, the use of SMR would solve the issue of resource dependence. A clear understanding about nutrient uptake and assimilation would encourage the replacement of peat by SMR for seedling culture of fragrant rosewood.

In this study, fragrant rosewood seedlings were cultured in different types of growing media with a range of SMR proportions used as a replacement for peat. We hypothesized that: (i) a low proportion of SMR, about 25% of total volume, would benefit fragrant rosewood seedlings with the highest nutrient uptake and assimilation, and (ii) excellent nutrient uptake and assimilation mostly accounted for the optimum performance of seedlings.

## Materials and methods

### Plant material and growth condition

Fragrant rosewood (*Dalbergia odorifera* T.C. Chen) seeds were obtained from mature trees from the Jing Mountain (28°08’ N, 120°38’E), Yongjia, Wenzhou city, Zhejiang province, China. It was stated by authors for the ethics that the land for field seed collection was issued by the authority permission of Zhejiang Institute of Subtropical Crops of Zhejiang Academy of Agricultural Sciences. Cleaned seeds were soaked in potassium permanganate solution (w/w, 0.5%) for 30 min and soaked in distilled water at indoor temperature for 12 h. Dead seeds were removed, and the remaining seeds were sown in sands. In May 2017, one month after sowing, germinated seedlings were transferred to embedded cavities in cultivation trays. Each tray had 32 cavities that were arranged with a 4×8 spacing, each cavity had a volume of 212 ml. Three seeds were planted at the growing media surface in one cavity. The experiment was finished in a growing chamber of an indoor laboratory, where temperature was controlled in a range between 18°C and 34°C and relative humidity (RH) was kept 70–90%. Seedlings were evenly exposed to lights from high-pressure sodium (HPS) lamps (Pudao Photoelectricity, Zhiluntuowei A&F S&T, Ltd., Changchun, China) in an 18-h photoperiod (06:00 am to 24:00 am) at the photosynthetic photon flux rate (PPFD) of 73 μmol m^-2^ s^-1^. More details about the lighting condition can be found in other studies [18,19]. Trays were placed in tanks that were watered twice a week. This irrigation methodology has been used for the culture of seedlings from several other woody plants [16,17,24].

### The property of spent mushroom residue

Spent mushroom residue was obtained from a local mushroom factory after the cultivation of *Pleurotus eryngii*. This mushroom is one of six widely produced species in China that has significant reserves of polysaccharides with specific beneficial enzyme profiles [25,26]. The process to dispose of SMR was adapted from the standard protocol to produce commercial substrates (Mashiro-Dust, Zhiluntuowei A&F S&T, Ltd., Changchun, China), which generally included peeling off the plastic coat, smashing to pieces, homogeneous mixing, dehydration, and sterilizing with ultraviolet rays. SMR was not composted in our study because it was suggested that fresh residue can be directly used in the growing media [11,12]. The components in the raw material of SMR included 20% wood bits, 20% cottonseed hull, 20% brans, 24% corncobs, 5% corn flour, 2% lime carbonate, 1% gypsum powder, and 8% bean pulp [12]. One 5-g sample of substrates was mixed in distilled water, mineral nitrogen (N) was termed as sum of ammonium N and nitrate N determined by a flow injection analysis system (Lachat Instruments, Hach CO., Loveland, USA). Another 5-g of substrate sample was extracted with 50 mL of potassium chloride (2 M) and shaken for 1 h. Available phosphorus (P) was determined using Inductively Coupled Plasma Optical Emission Spectrometry (ICP-OES) (Thermo Fisher Scientific, Waltham, MA, USA).

### Experimental design and treatment description

Five proportions of SMR as replacement of peat were employed in the growing media, 0% (control), 25% (75% peat), 50% (50% peat), 75% (25% peat), and 100% (no peat) (v/v). Basic chemical properties in these five types of substrates are shown in Table 1. A total of 32 seedlings were assigned as one sampling unit per tray. Three trays were arranged as replicates in one treatment. All trays of seedlings were placed to evenly receive HPS lighting with a random plots design. Trays were rearranged to be moved twice a week after seedlings were watered.

**Table 1:** Chemical property of substrate composed with un-composted spent mushroom residue (USMR) and peat in different ratios for the culture of fragrant rosewood (*Dalbergia odorifera* T.C. Chen) seedlings under plant-factory conditions.

Parameter	USMR component ratio				
	0%	25%	50%	75%	100%
Ammonium N ^1^ (mg kg^-1^)	127.07±7.49a ^2^	120.55±0.57a	113.39±1.51a	74.78±8.92b	22.25±3.20c
Nitrate N (mg kg^-1^)	154.61±9.93a	139.43±5.44a	50.91±5.32b	39.36±7.66b	13.40±6.86c
Available P ^2^ (mg kg^-1^)	236.62±21.66e	365.48±3.73d	512.02±9.16c	636.13±11.09b	906.67±15.88a
pH value	4.33±0.10d	4.52±0.02cd	4.69±0.02c	5.17±0.17b	5.70±0.03a
EC ^4^ (dS m^-1^)	0.35±0.13c	1.00±0.11bc	1.55±0.19b	2.44±0.36a	2.67±0.17a

^1^ N, nitrogen;

^2^ Different letters in a row indicate significant difference among USMR ratios;

^3^ P, phosphorus;

^4^ EC, electrical conductance.

### Sampling and chemical analysis

**Fig 1:**
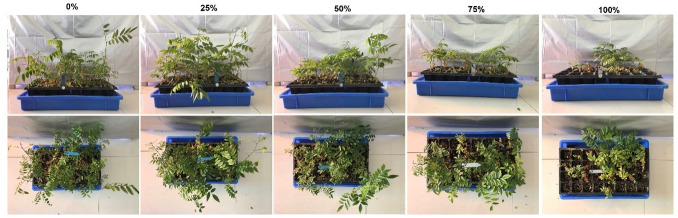
The outcome of performance of fragrant rosewood (*Dalbergia odorifera* T.C. Chen) seedlings subjected to growing media with spent mushroom residue (SMR) in volumetric proportions of 0%, 25%, 50%, 75%, and 100%.

We raised seedlings for 4.5 months until October 2017 when they were scheduled to be sampled (Fig 1). Eight seedlings were sampled from one tray and their average was assigned as a basic analysis unit. Four were used for drying and the other four kept in a freezer at -18°C. Sampled seedlings were divided into shoot and root parts, the shoot part was used for measuring height and root-collar diameter (RCD). Roots were washed and divided into coarse (diameter ≥1 mm) and fine roots (diameter <1 mm). Both shoot and root parts were dried in an oven at 70°C for three days and then weighted for dry mass. Dried samples were ground to pass 1-mm sieve for further analysis of non-structural carbohydrates (NSCs) and nutrients.

The colorimetric method was adapted to determine soluble sugars and starch concentrations using a spectrophotometer (UV-Visible 8453, Agilent Technologies Inc., Santa Clara, CA, USA) at 490 nm. A 0.5-g sample was dissolved in 50 mL of distilled water and boiled for two hours. The cooled supernatant was collected to a volume of 30 mL for the determination of soluble sugar concentration. The residual was washed and oven-dried at 70°C till no moist remained. Dried residue was moved to 10 mL of 3% (v/v) hydrochloric acid, extracted, and maintained in boiling water for eight hours. Cooled residue was centrifuged and collected to obtain 1mL of supernatant added to 28% phenol (w/w) and 5 mL of sulfuric acid. Once again, starch was determined using a spectrophotometer at 490 nm.

Another 0.2 g of dried sample was digested in 5 mL of solution mixed by hydrogen peroxide and sulfuric acid. Digested soluble was diluted to 50 mL and used for measuring N and P concentrations.

The remaining four seedlings per tray were used to determine enzyme activities of glutamine synthetase (GS) and acid phosphatase (ACP) according to the method of Wei et al. [27]. For GS, the absorbance of the glutamyl-γ-hydroxamate was measured at 540 nm. Protein concentration was determined by the procedure of Coomassie Brilliant blue G-250 [16,27]. For ACP, the ground sample was centrifuged at 10000 g for 10 min and the supernatant was moved to 10 mL of solution. Absorbance of the reaction mixture was measured spectrophotometrically at 405 nm [16,27].

### Parameter calculation and statistic analysis

Seedling quality was evaluated by the Dickson index model [28]:

$$DQI=\frac{TB}{\frac{Height}{RCD}+\frac{SB}{RB}}$$ (1)

where *DQI* is the Dickson quality index; *TB*, *SB*, and *RB* stand for biomass in whole plant, shoot, and root parts, respectively. Nutrient utilization index was calculated as shoot biomass divided by shoot nutrient concentrations according to a model that was used by Hawkins [29]. Utilization index for N and P was differentiated by N (*NUI*) and P concentrations (*PUI*), respectively.

Data were analyzed by SAS software (ver. 9.4, SAS Institute, Cary, NC, USA). Data were tested for normality, no transformation was necessary. Data were tested by the one-way analysis of variance (ANOVA) to detect the effect of five treatments of growing media on seedling parameters. When a significant effect was indicated by ANOVA (*P*≤0.05), means were compared and arranged according to the Tukey test (α = 0.05). Pearson correlation was employed to detect the relationship between any pair of variables about substrate property and seedling parameters. In order to diagnose nutritional status in plants subjected to different substrates, vector analysis was employed at the end of the experiment using the control as the reference. Nutrient content and concentration and biomass were standardized to the three-dimensional nomograph on x-, y-, and z-axes, respectively. The standardization was performed as transformation to a value in the range from 0 to 100 with that in the control as the value of 0. Vector shifts and the corresponding interpretations were adapted from Salifu and Timmer [30].

## Results

### Substrate property

With the increase of SMR proportion in growing media, both ammonium N and nitrate N concentrations showed a trend of decline (Table 1). Compared to the control, ammonium N concentration was not significantly lower until the 75% and 100% SMR proportion treatments; nitrate N concentration decreased beginning in the 50% SMR proportion treatment. In contrast, available P concentration was higher in all growing media with SMR than in the control. Substrate pH value was also increased, which was significantly higher in the 50% SMR proportion treatment than the control. Electrical conductance (EC) value rose with the increase of SMR proportion in growing media, EC in the 25% treatment was not statistically different from that in the control.

### Seedling growth, biomass, and quality assessment

As shown in Fig 1, fragrant rosewood seedlings did not show any negative symptoms in the control and the 25% SMR proportion treatment. In the 50% treatment, some seedlings showed wilted stems and yellowed leaves, while in the 75% treatment the number of seedlings with these negative symptoms increased to about 1/3 per tray. Approximately half of seedlings died in the 100% SMR proportion treatment, where the living seedlings overwhelmingly showed unhealthy appearances.

Both seedling height and RCD showed a trend of decline with the increase of volumetric proportion of SMR in growth media (*F*_4,10_ = 10.43; *P* = 0.0014) (Table 2). Compared to seedling height in the control, that in the 75% and 100% SMR proportion treatments decreased by 71% and 43%, respectively; RCD decreased by 16% and 19%, respectively.

**Table 2:** Growth, biomass accumulation, and quality evaluation of fragrant rosewood (*Dalbergia odorifera* T.C. Chen) seedlings in response to the substrates composed with un-composted spent mushroom residue (USMR) and peat in different ratios under plant-factory conditions.

Parameter	USMR component ratio				
	0%	25%	50%	75%	100%
Height (cm)	28.30±2.41ab ^1^	31.36±3.29a	26.07±3.42ab	20.10±1.78bc	16.13±4.23c
RCD (cm) ^2^	0.31±0.02ab	0.36±0.00a	0.30±0.04ab	0.26±0.01b	0.25±0.03b
Shoot biomass (g)	3.04±0.25ab	3.81±0.18a	2.40±0.56b	1.38±0.10c	1.16±0.36c
Root biomass (g)	0.37±0.07b	0.70±0.05a	0.38±0.12b	0.21±0.02b	0.19±0.03b
R/S ^3^	0.12±0.02a	0.18±0.02a	0.16±0.03a	0.15±0.02a	0.17±0.03a
*DQI* ^4^	34.41×2.23b	48.86×5.79a	30.32±8.19bc	19.11±0.64c	19.19±2.58c
*NUI* ^5^	0.18±0.04a	0.18±0.01a	0.16±0.10a	0.08±0.02a	0.07±0.02a
*PUI* ^6^	0.49±0.01ab	0.62±0.02a	0.36±0.10b	0.20±0.02c	0.19±0.04c

^1^ Different letters in a row indicate significant difference among USMR ratios;

^2^ RCD, root-collar diameter;

^3^ R/S, root to shoot biomass ratio;

^4^ *DQI*, Dickson quality index;

^5^ *NUI*, nitrogen utilization index;

^6^ *PUI*, phosphorus utilization index.

The proportion of SMR in growing media had a significant effect on biomass in shoot (*F*_4,10_ = 30.67; *P*<0.0001) and root (*F*_4,10_ = 23.00; *P*<0.0001), both of which declined with the increase of SMR proportion (Table 2). Compared to shoot biomass in the control, that in 75% and 100% SMR proportion treatments decreased by 100% and 62%, respectively. Compared to root biomass in the control, only that in the 25% SMR proportion treatment was higher by 88% with all the remaining treatments resulting in insignificant difference. The proportion of SMR in growing media had no effect on root to shoot biomass ratio (R/S) (*F*_4,10_ = 2.08; *P* = 0.1582).

The proportion of SMR in growing media had a significant effect on *DQI* (*F*_4,10_ = 18.21; *P* = 0.0001). Compared to *DQI* in the control, that in the 25% proportion treatment was higher by 42%, while that in the 75% and 100% treatments were lower by 45% and 44%, respectively (Table 2).

As shown in Table 2, the proportion of SMR in growing media had no effect on *NUI* (*F*_4,10_ = 3.25; *P* = 0.0593), but the effect on *PUI* was significant (*F*_4,10_ = 33.93; *P*<0.0001). Compared to the control, the 25% and 50% SMR proportion treatments did not have significant difference of *PUI*, which was lower by 100% and 62% in the 75% and 100% treatments, respectively.

### Nutrient concentration and content in plants

**Fig 2:**
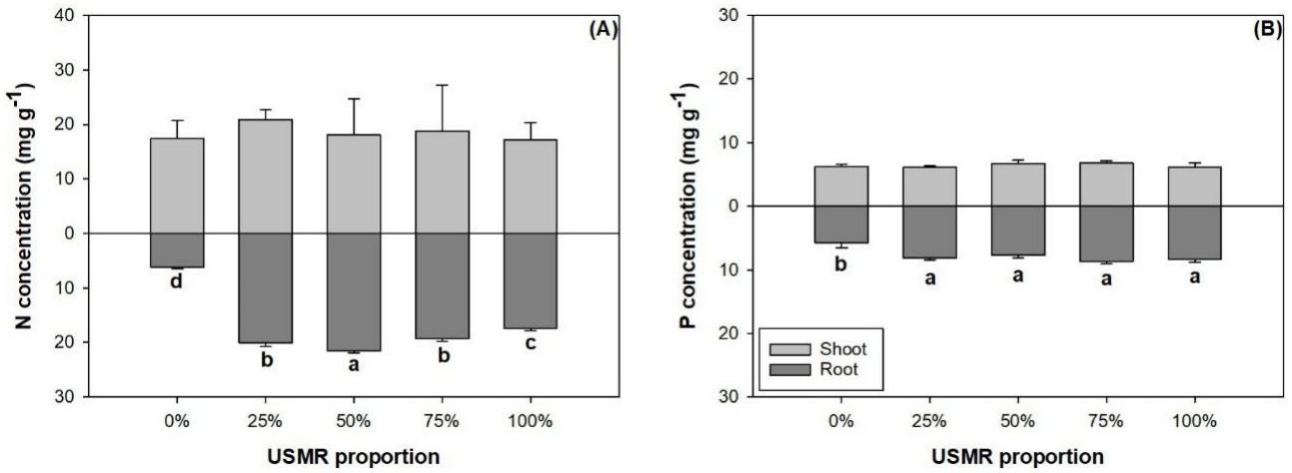
Nitrogen (N) (A) and phosphorus (P) (B) concentrations in shoot and root parts of fragrant rosewood (*Dalbergia odorifera* T.C. Chen) seedlings subjected to growing media with un-composted spent mushroom residue (USMR) in volumetric proportions of 0%, 25%, 50%, 75%, and 100%.

The proportion of SMR in growing media had no effect on N (*F*_4,10_ = 0.40; *P* = 0.8061) and P concentrations (*F*_4,10_ = 1.62; *P* = 0.2436) in the shoot part (Fig 2A and 2B). Instead, the effect of SMR proportion was significant on root N (*F*_4,10_ = 489.06; *P*<0.0001) and P concentrations (*F*_4,10_ = 14.97; *P* = 0.0003). With the increase of SMR proportion, root N concentration increased from the control to the 50% treatment, then declined to the 100% treatment (Fig 2A). Root P concentration was higher by 33–51% in growing media with SMR than that in the control (Fig 2B).

**Fig 3:**
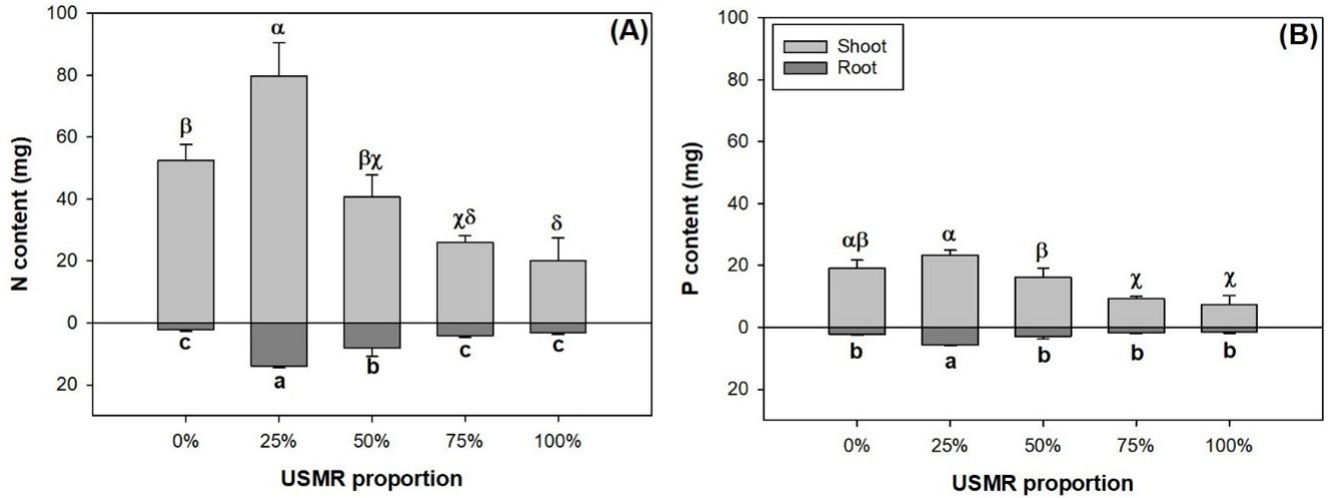
Nitrogen (N) (A) and phosphorus (P) (B) contents in shoot and root parts of fragrant rosewood (*Dalbergia odorifera* T.C. Chen) seedlings subjected to growing media with un-composted spent mushroom residue (USMR) in volumetric proportions of 0%, 25%, 50%, 75%, and 100%.

The proportion of SMR in growing media had a significant effect on N (*F*_4,10_ = 30.53; *P*<0.0001) and P contents (*F*_4,10_ = 21.76; *P*<0.0001) in the shoot part (Fig 3A and 3B). Shoot N content was highest in the 25% SMR proportion treatment (Fig 3A). Compared to that in the control, shoot N content was lower in the 75% and 100% treatments by 100% and 62%, respectively Shoot P content was not statistically different in the 25% SMR proportion treatment compared to that in the control (Fig 3B). Compared to the control, the 75% and 100% treatments resulted in lower shoot P content by 51% and 62%, respectively. The proportion of SMR in growing media had a significant effect on N (*F*_4,10_ = 38.72; *P*<0.0001) and P contents (*F*_4,10_ = 33.82; *P*<0.0001) in the root part (Fig 3A and 3B). Among all treatments, both root N and P contents were highest in the 25% SMR proportion treatment.

### Nutrient status diagnosis

**Fig 4:**
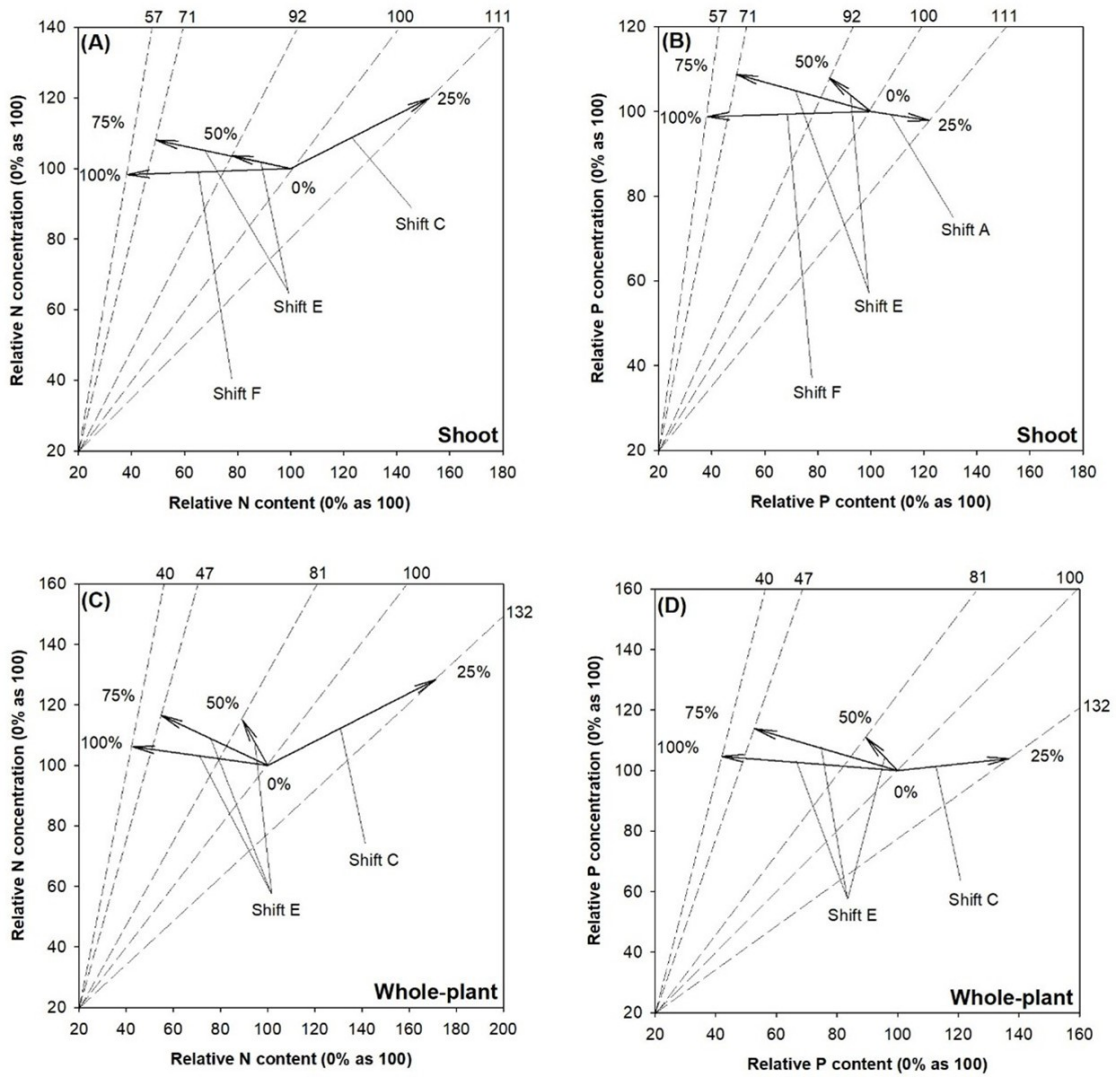
Vector diagnosis of nitrogen (N) (left) and phosphorus (P) (right) statuses in shoot (upper) and whole-plant (lower) of fragrant rosewood (*Dalbergia odorifera* T.C. Chen) seedlings subjected to growing media with un-composted spent mushroom residue (USMR) in volumetric proportions of 0%, 25%, 50%, 75%, and 100%. Shift A, nutrient dilution; Shift C, alleviation of nutrient deficiency; Shift E & F, excessive nutrient toxicity. Shifts and interpretations are adapted from Salifu and Timmer [30].

Relative to controlled seedlings, those in the 50% and 75% SMR treatments were diagnosed as nutrient (both N and P) excess possibly caused by toxicity accumulation (Fig 4A and 4B). Seedlings in the 100% treatment showed a relative nutrient excess possibly caused by nutritional antagonism. Controlled seedlings showed symptoms of N deficiency due to limitation of biomass production, which was alleviated by the 25% SMR proportion treatment (Fig 4A). The 25% treatment also resulted in P dilution compared to the control due to increases in biomass and P content but a decline of P concentration (Fig 4B).

Whole-plant N status was similar to that in the shoot part (Fig 4C). Whole-plant P status in the control was diagnosed as a deficiency, which was alleviated in growing media with 25% SMR proportion (Fig 4B).

### Non-structural carbohydrate concentration

**Fig 5:**
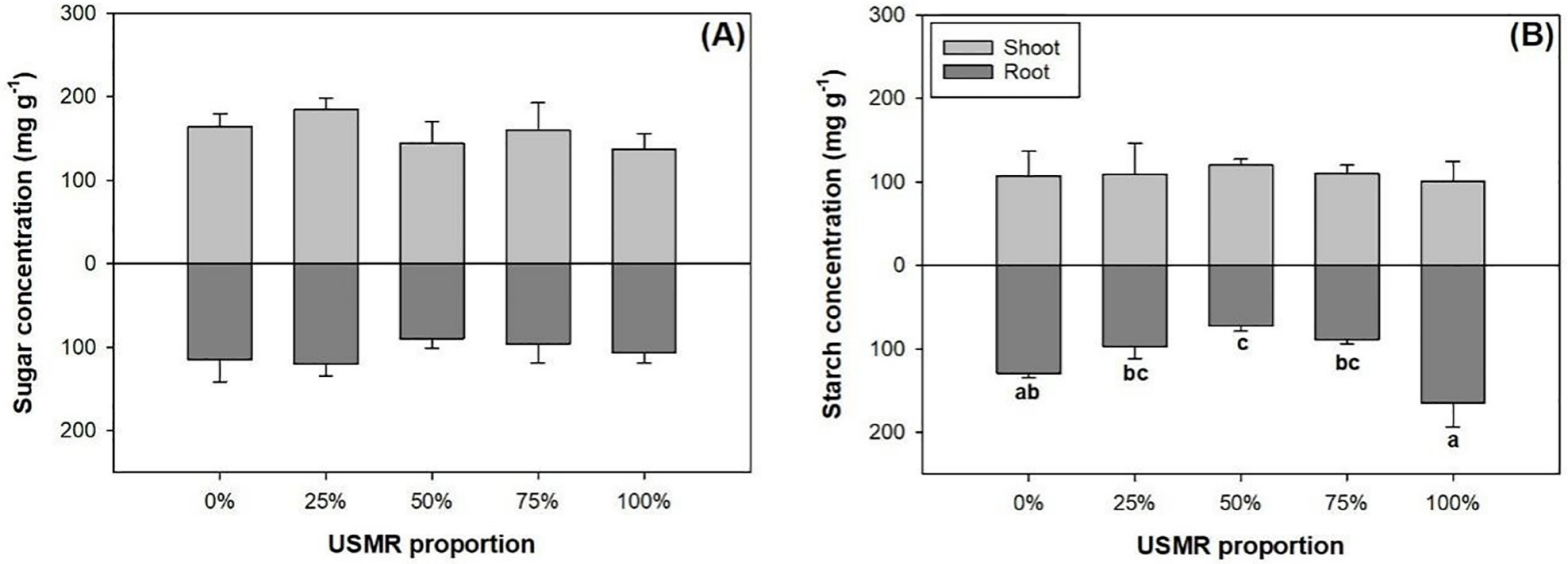
Soluble sugars (A) and starch (B) concentrations in shoot and root parts of fragrant rosewood (*Dalbergia odorifera* T.C. Chen) seedlings subjected to growing media with un-composted spent mushroom residue (USMR) in volumetric proportions of 0%, 25%, 50%, 75%, and 100%.

The proportion of SMR in growing media had no effect on soluble sugar (*F*_4,10_ = 0.46; *P* = 0.7661) and starch concentrations (*F*_4,10_ = 0.21; *P* = 0.9250) in the shoot part (Fig 5A and 5B). The proportion of SMR in growing media also had no effect on soluble sugar concentration (*F*_4,10_ = 1.31; *P* = 0.3299) in the root part (Fig 5C). However, the proportion of SMR in growing media had a significant effect on starch concentration (*F*_4,10_ = 16.32; *P* = 0.0002) in the root part (Fig 5C). Compared to the control, the 50% SMR proportion treatment resulted in lower root starch concentration by 44% (Fig 5D).

### Nutrient assimilation enzyme activities

**Fig 6:**
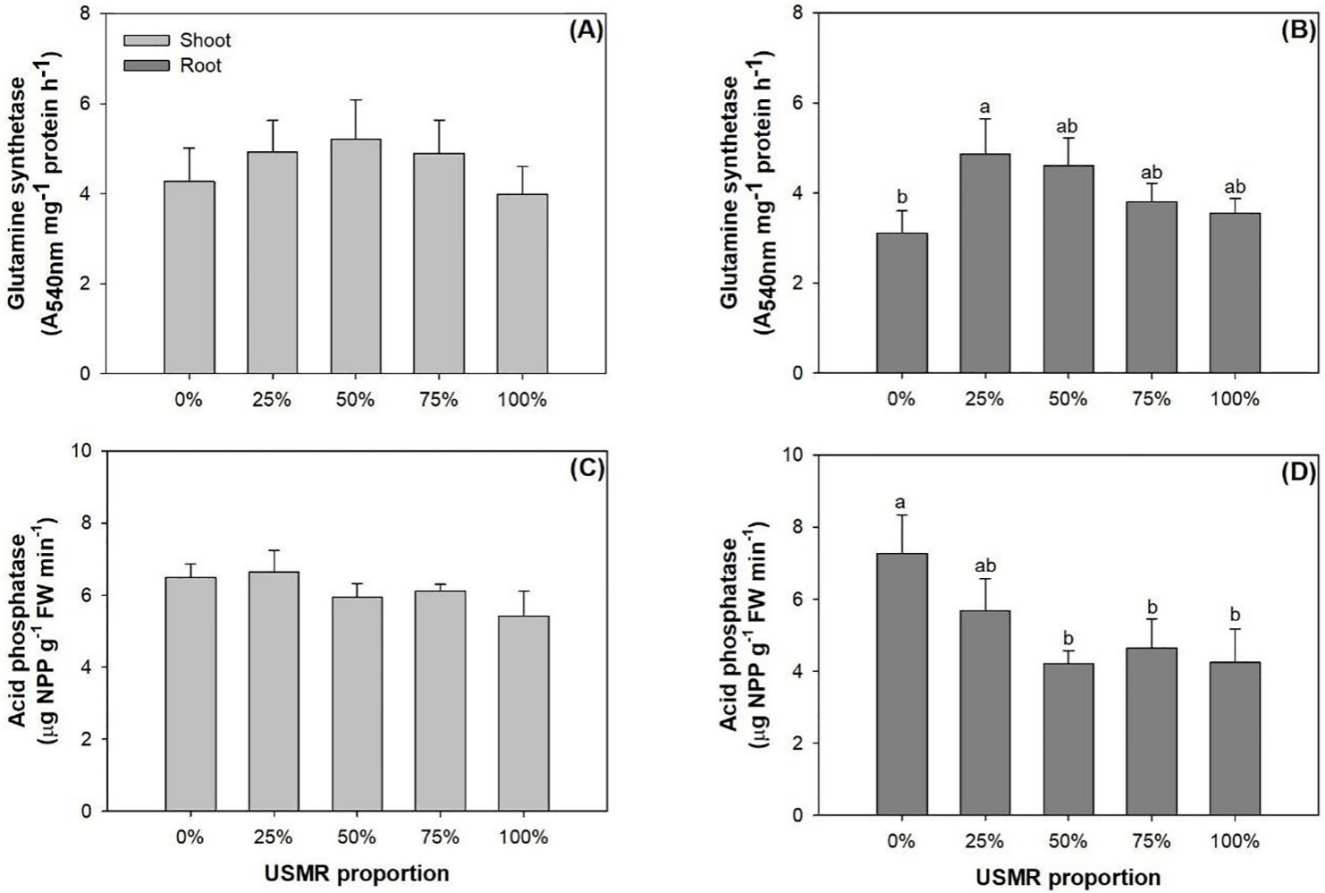
Activities of glutamine synthetase (upper) and acid phosphatase (lower) in shoot (left) and root (right) parts of fragrant rosewood (*Dalbergia odorifera* T.C. Chen) seedlings subjected to growing media with un-composted spent mushroom residue (USMR) in volumetric proportions of 0%, 25%, 50%, 75%, and 100%.

The proportion of SMR in growing media had no effect on enzyme activities for GS (*F*_4,10_ = 1.26; *P* = 0.3470) and ACP (*F*_4,10_ = 2.64; *P* = 0.0968) in the shoot part (Fig 6A and 6C). However, the proportion of SMR in growing media had a significant effect on GS activity in the root part (*F*_4,10_ = 4.66; *P* = 0.0221). Compared to the control, the 25% SMR proportion treatment resulted in a higher GS activity by 56% (Fig 6B). The proportion of SMR in growing media also had a significant effect on ACP activity in the root part (*F*_4,10_ = 6.29; *P* = 0.0085). Compared to AC activity in the control, that in the 50%, 75%, and 100% treatments was lower by 36–42% (Fig 6D).

### Correlation between substrates and seedlings

As is shown in Table 3, available P had a negative correlation with growth and biomass in fragrant rosewood seedlings, but their correlation with mineral N was positive. Available P also had a negative correlation with shoot N and P contents and, again, the correlation with mineral N positive. Available P had a negative relationship with ACP activity, whose correlation with mineral N was positive. Both pH and EC values had a negative correlation with growth, biomass, shoot N and P contents, and ACP activities, but the correlation was positive with root P concentration.

**Table 3:** Pearson correlation between substrate property indices and growth and nutritional parameters in fragrant rosewood (*Dalbergia odorifera* T.C. Chen) seedlings cultured with the substrate with composed un-composted spent mushroom residue (USMR) and peat in different ratios under plant-factory conditions (*n* = 15).

Seedling parameter	Substrate property indices				
	Available P	Ammonium N	Nitrate N	pH value	EC value ^1^
Height	***R* = -0.8406**	***R* = 0.8272**	***R* = 0.7780**	***R* = -0.8311**	***R* = -0.7828**
	***P*<0.0001** ^2^	***P* = 0.0001**	***P* = 0.0006**	***P* = 0.0001**	***P* = 0.0006**
RCD ^3^	***R* = -0.7139**	***R* = 0.7041**	***R* = 0.7143**	***R* = -0.7125**	***R* = -0.7026**
	***P* = 0.0028**	***P* = 0.0034**	***P* = 0.0028**	***P* = 0.0029**	***P* = 0.0035**
Shoot biomass	***R* = -0.8384**	***R* = 0.8048**	***R* = 0.8725**	***R* = -0.8402**	***R* = -0.8547**
	***P*<0.0001**	***P* = 0.0003**	***P*<0.0001**	***P*<0.0001**	***P*<0.0001**
Root biomass	***R* = -0.6391**	***R* = 0.6629**	***R* = 0.6822**	***R* = -0.6633**	***R* = -0.6196**
	***P* = 0.0103**	***P* = 0.0071**	***P* = 0.0051**	***P* = 0.0070**	***P* = 0.0138**
Shoot N conc. ^4^	*R* = -0.1433	*R* = 0.2302	*R* = 0.1895	*R* = -0.2241	*R* = -0.2139
	*P* = 0.6105	*P* = 0.4092	*P* = 0.4989	*P* = 0.4221	*P* = 0.4440
Root N conc.	*R* = -0.4699	*R* = -0.2451	***R* = -0.5618**	*R* = 0.3663	***R* = 0.5602**
	*P* = 0.0772	*P* = 0.3786	***P* = 0.0293**	*P* = 0.1793	***P* = 0.0299**
Shoot N cont. ^5^	***R* = -0.7291**	***R* = 0.7359**	***R* = 0.8010**	***R* = -0.7471**	***R* = -0.7611**
	***P* = 0.0020**	***P* = 0.0018**	***P* = 0.0003**	***P* = 0.0014**	***P* = 0.0010**
Root N cont.	*R* = -0.2989	*R* = 0.4195	*R* = 0.3098	*R* = -0.3740	*R* = -0.2472
	*P* = 0.2792	*P* = 0.1196	*P* = 0.2612	*P* = 0.1696	*P* = 0.3745
Shoot P conc.	*R* = 0.0673	*R* = 0.0351	*R* = -0.3187	*R* = 0.0268	*R* = 0.1977
	*P* = 0.8116	*P* = 0.9013	*P* = 0.2469	*P* = 0.9246	*P* = 0.4801
Root P conc.	***R* = 0.6419**	***R* = -0.5328**	***R* = -0.6187**	***R* = 0.6011**	***R* = 0.7290**
	***P* = 0.0099**	***P* = 0.0409**	***P* = 0.0139**	***P* = 0.0178**	***P* = 0.0020**
Shoot P cont.	***R* = -0.8442**	***R* = 0.8227**	***R* = 0.8449**	***R* = -0.8523**	***R* = -0.8496**
	***P*<0.0001**	***P* = 0.0002**	***P*<0.0001**	***P*<0.0001**	***P*<0.0001**
Root P cont.	*R* = -0.4787	***R* = 0.5315**	***R* = 0.5357**	***R* = -0.5192**	*R* = -0.4380
	*P* = 0.0710	***P* = 0.0414**	***P* = 0.0396**	***P* = 0.0473**	*P* = 0.1025
Shoot sugar conc.	*R* = -0.2672	*R* = 0.2561	*R* = 0.3335	*R* = -0.2769	*R* = -0.3507
	*P* = 0.3356	*P* = 0.3569	*P* = 0.2245	*P* = 0.5461	*P* = 0.2000
Root sugar conc.	*R* = 0.2581	*R* = 0.1408	*R* = 0.4155	*R* = -0.1309	*R* = -0.3561
	*P* = 0.3529	*P* = 0.6168	*P* = 0.1235	*P* = 0.6420	*P* = 0.1927
Shoot starch conc.	*R* = -0.1062	*R* = 0.1062	*R* = 0.0555	*R* = -0.1694	*R* = -0.0973
	*P* = 0.7064	*P* = 0.7064	*P* = 0.8442	*P* = 0.5461	*P* = 0.7302
Root starch conc.	*R* = 0.3800	***R* = -0.5537**	*R* = -0.1182	*R* = -0.4604	*R* = 0.1388
	*P* = 0.1624	***P* = 0.0322**	*P* = 0.6750	*P* = 0.0842	*P* = 0.6217
Shoot GS ^6^	*R* = -0.1860	*R* = 0.3597	*R* = -0.0165	*R* = -0.2241	*R* = -0.0964
	*P* = 0.5069	*P* = 0.1879	*P* = 0.9535	*P* = 0.4220	*P* = 0.7327
Root GS	*R* = -0.0830	*R* = 0.2421	*R* = 0.0514	*R* = -0.1817	*R* = -0.0804
	*P* = 0.7687	*P* = 0.3846	*P* = 0.8557	*P* = 0.5170	*P* = 0.7758
Shoot ACP ^7^	***R* = -0.6488**	***R* = 0.6453**	***R* = 0.6116**	***R* = -0.5894**	***R* = -0.5576**
	***P* = 0.0089**	***P* = 0.0094**	***P* = 0.0154**	***P* = 0.0208**	***P* = 0.0308**
Root ACP	***R* = -0.6751**	***R* = 0.5165**	***R* = 0.7692**	***R* = -0.5917**	***R* = -0.6401**
	***P* = 0.0058**	***P* = 0.0487**	***P* = 0.0008**	***P* = 0.0201**	***P* = 0.0102**

^1^ EC, electrical conductance;

^2^ Bold values indicate significant correlation;

^3^ RCD, root-collar diameter;

^4^ conc., concentration;

^5^ cont. content;

^6^ GS, glutamine synthetase;

^7^ ACP, acid phosphatase.

## Discussion

It is widely recognized that high salinity is the critical factor of SMR properties that causes stress to plants [6,10–12]. In our study, the 100% SMR proportion treatment involved growing media with no peat where seedlings showed heavy mortality and twig withered. Although EC value in the 100% SMR proportion treatment (2.67±0.29 dS m^-1^) was higher than that in the control and SMR-mixed growing media in a proportion from 25% to 50%, our highest EC value was not as high as that in the substrates of SMR and Sphagnum peat (3.06 dS m^-1^) [10] and in the substrates mostly mixed with *Agaricus bisporus* SMR and peat (2.66–5.90 dS m^-1^) [6,11]. However, our EC value appeared to be higher than that in the growth media with *Pleurotus ostreatus* SMR (0.51–1.03 dS m^-1^) [6] and *Lentinula edodes* SMR (2.54 dS m^-1^) [11]. With the same mushroom cultivation, our EC value was close to that from *Pleurotus eryngii* SMR (2.74 dS m^-1^) used for the culture of pepper plants [12]. Regarding results about negative relationship between substrate salinity and plant variables, high salinity is also the cause in SMR that shaped stresses on fragrant rosewood seedlings.

Due to the negative property of high salinity in SMR, a high proportion would generate a high risk to plant growth; hence peat in the growing media cannot be fully replaced by SMR. Medina et al. [6] raised tomato, curgetti, and pepper plants in two types of SMR growing media and revealed that fresh weight of seedlings decreased with the increase of SMR proportion. Zhu et al. [12] also found that pepper seedlings showed negative responses of growth and biomass accumulation to the increase of SMR proportion. In our study, shoot growth and biomass accumulation in both shoot and root parts all had a negative relationship with EC value, but high SMR substrates did not actually reduce diameter growth and failed to affect biomass allocation to the root part. The most significant negative impact of the 100% SMR treatment came from the decline of seedling height, and accordingly, the shoot biomass. This was why *DQI* also decreased in the growing media without any peat. Therefore, the most remarkable influence was the negative impact on shoot elongation and its further impact on biomass production without any impact on biomass allocation to roots. As fragrant rosewood is a woody plant, its resistance to negative properties in SMR is different from that in crop plants. With attention to SMR replacement of peat being developed by people working horticultural plant production, it is essential for more woody plants y to be tested for their response to high SMR proportion in the growing media in future works.

In our study, the volumetric proportion of SMR in growing media had no effect on seedling N concentration, *NUI*, and GS activity. These together suggest that the failure to influence N assimilation further imposed the null effect on N utilization. GS plays an important role in N assimilation with regard to synthesizing NH_4_^+^ into glutamine [31]. Neither ammonium N nor nitrate N in growing media had any relationship with shoot N concentration or leaf GS activity. The mineral N in SMR substrates cannot be assimilated as glutamine in leaves. Most SMR treatments resulted in excessive N toxicity mainly due to decreased biomass relative to the 100% peat substrate. Our results concur with those in previous studies, which together demonstrate inhibition of N assimilation pathway through GS activity caused N toxicity [32,33]. The salinity in SMR in proportions higher than 25% caused the toxicity and further limited N assimilation in leaves. Because the whole-plant N status was diagnosed to suffer N toxicity as N status in shoot, higher root N concentration was a symptom of deeper toxicity that explains the negative nitrate N concentration in SMR substrates with root N concentration. Only the 25% SMR proportion in the growing media was able to elicit positive response of N status by alleviating N deficiency in controlled seedlings. This was because only seedlings in the 25% SMR proportion obtained an increase in biomass compared to those in 100%-peat substrates. The optimum proportion of SMR with 25% replacement of peat in growing media in our study concur with that found on tomato and pepper seedlings [12,15].

Shoot P concentration also showed no response to SMR-mixed substrates, nor did leaf ACP activity. We found that shoot P concentration had no relationship with available P concentration in growing media but the relationship with leaf ACP activity was negative. This is reasonable because the limit of ACP activity occurred only when P availability tended to be lowered [34]. In contrast, root P concentration can be promoted by SMR-mixed substrates which benefitted from the positive relationship with P availability. Again, root ACP activity was also negatively related to P availability. Because whole-plant P fell in a status of excessive toxicity, the positive relationship between EC and root P concentration meant that high salinity aggravated P toxicity. The negative relationship between salinity and ACP activity concur to previous evidence [35]. Because the 25% SMR treatment increased biomass, shoot P concentration was over diluted relative to the control, but root P concentration was promoted due to P uptake by roots.

Neither soluble sugars nor starch had any relationship with SMR-mixed substrates. Therefore, the change of root starch concentration among growing media resulted from inherent allocation and consumption instead of exogenous stimulation. We found that root starch concentration showed an inverse response to SMR treatments relative to root N concentration. This suggests that the reduction of starch concentration was used for N absorption in roots [36]. No response of soluble sugar concentration was found to SMR growing media which could result from hydrolysis from starch. More evidence is needed to detect non-structural carbohydrates in more species subjected to SMR substrates.

## Conclusion

Un-composted SMR can be used to replace some peat in the growing media for the culture of fragrant rosewood seedlings. The chemical property in substrates showed gradient change in response to the increase of volumetric proportion of SMR, which suggested relationship with seedling growth and P assimilation along the SMR-proportion gradient. Overall, the involvement of SMR in substrates did not cause much negative impact on seedling growth and biomass except for some suppression in height growth in high SMR proportions over 75%. Most SMR-mixed substrates induced N and P toxicities due to decline of biomass caused by salinity stress, leaving the proportion of 25% SMR in growing media as the optimum for biomass accumulation and deficiency alleviation. Overall, we conclude that fresh SMR has good potential to be used in growing media to replace 25% of peat for the culture of fragrant rosewood seedlings. Nutrient uptake and assimilation in this study can be tested in future work on more species of highly-valued woody plants.

## Supporting information

S1 Data (RAR)
